# The complete mitochondrial genome of the Dongan black chicken and its phylogenetic analyses

**DOI:** 10.1080/23802359.2018.1521306

**Published:** 2018-10-08

**Authors:** Si-Min Peng, Qian Lin, Gui-Tao Jiang, Ying-Hui Li, Qiu-Zhong Dai, Xi He, Hai-Feng Yan

**Affiliations:** a College of Food Science and Technology, Hunan Agricultural University, Changsha, PR China;; b Institute of Bast Fiber Crops, Chinese Academy of Agricultural Sciences, Changsha, PR China;; c Research Department of Animal Nutrition and Poultry, Hunan Institute of Animal and Veterinary Science, Changsha, PR China;; d College of Animal Science and Technology, Hunan Agricultural University, Changsha, PR China

**Keywords:** *Gallus gallus domesticus*, Dongan black chicken, mitochondrial, phylogenetic analyses

## Abstract

Dongan black chicken (*Gallus gallus domesticus*, DBC) is one of the famous native breed of Hunan province in China. The complete mitochondrial genome sequence plays an important role in the accurate determination of phylogenetic relationships among metazoans. It is the first time that the complete mitochondrial genome sequence of the DBC was reported. The total length of the mtDNA is 16,785 bp, It contains the typical structure, including 22 transfer RNA genes, 2 ribosomal RNA genes, 13 protein-coding genes and 1 non-coding control region (D-loop region). The overall composition of the mtDNA was estimated to be 30.27% for A, 23.78% for T, 32.46% for C, and 13.49% for G. Phylogenetic analyses using N-J computational algorithms showed that the analyzed 21 Galliformes species are divided into four major clades: Phasianidae, Numidiidae, Odontophoridae, and Megapodiidae. In addition, our work confirmed that DBC and Taoyuan chicken (TYC) have a close genetic relationship with fellow tribal members Xuefeng black-boned chicken (XBC) and Huang Lang chicken (HLC). Meanwhile, we also found that DBC and TYC have highly similar genetic relationship. This work will provide an important data set for the study of genetic mechanism of chicken in Hunan province.

Domestic chicken (*Gallus gallus domesticus*) play a key role in the agricultural and economic sectors in Hunan Province of China. There are numerous domestic chicken breeds in Hunan Province including Taoyuan chicken (TYC), Huang Lang chicken (HLC), Xuefeng black-boned chicken (XBC) and Dongan black chicken (DBC) (Yu et al. 2016; Liu et al. 2016a; 2016b). However, a better characterization of the genetic diversity of these domestic chicken breeds is in urgent need to increase the conservational application for them in Hunan province. In addition, only the complete mitochondrial genome of DBC has not been reported. The features of DBC is strong adaptability, good stress resistance and good meat quality (Chen 2013). In this study, we determined the complete mitochondrial genome of DBC, and the adult individuals of DBC were collected at its originally breeding farm in Dongan County (26.41′N and 111.28′E), Hunan Province, China on September 2015. And the specimens were stored at −70 °C in our laboratory (Collaborative Innovation Center for Livestock and Poultry Safety Production, Hunan Agricultural University). Total genomic DNA was extracted from the thorax muscle of a single individual using the EasyPure Kit of Genomic DNA (Transgen Biotech, Beijing, China). The whole mitochondrial genome was amplified with nine pairs of primers and sequenced by BioSune Biotech (Shanghai, China). DNA sequence was analyzed using DNAStar.Lasergene.v7.1 software (Madison, WI), tRNA Scan-SE1.21 software (Lowe and Eddy 1997) and DOGMA software (Wyman et al. 2004).

The total length of the mtDNA is 16,785 bp. It contains the typical structure, including 22 transfer RNA genes, 2 ribosomal RNA genes, 13 protein-coding genes, and 1 non-coding control region (D-loop region) (GenBank accession No. KM886936). The overall composition of the mtDNA was estimated to be 30.27% for A, 23.78% for T, 32.46% for C, and 13.49% for G. All the protein initiation codons are ATG, except for COX1 is GTG. All these genes had 15 spaces in the length of 1–9 bp and had 7 overlaps in the length of 1–10 bp. These genes had four types of termination codons, including TAG, TAA, AGG, and an incomplete termination codon ‘‘T––’’, ‘‘T––’’ is the 5' terminal of the adjacent gene (Anderson et al. 1981). Among 13 protein-coding genes, the longest one was ND5 gene (1818 bp), which was located between the tRNA^Leu^ and Cytb, and the shortest one was ATPase8 gene (165 bp), which was located between the tRNA^Lys^ and ATPase6.

Phylogenetic analysis was performed using the complete mitochondrial DNA sequences of 21 Galliformes. Each of the sequence datasets was aligned by ClustalX (Thompson et al. 1997) and analyzed by neighbor-joining (N-J) in MEGA 4.0 (Tamura et al. 2007), and bootstrap analysis was performed with 100 replications. An N-J tree showed that the analyzed species are divided into four major clades (Figure 1). Phasianidae makes up the first lineage, which is sister to the second group, Numidiidae; Odontophoridae forms the third group and is sister to Phasianidae and Numidiidae. The lineage consisting of these three groups, in turn, is sister to the fourth clade, Megapodiidae. In addition, our work confirmed that DBC and TYC have a close genetic relationship with fellow tribal members XBC and HLC. Meanwhile, we also found that DBC and TYC have highly similar genetic relationship which is consistent with those as reported previously (Zhao et al. 2016).

**Figure 1.**
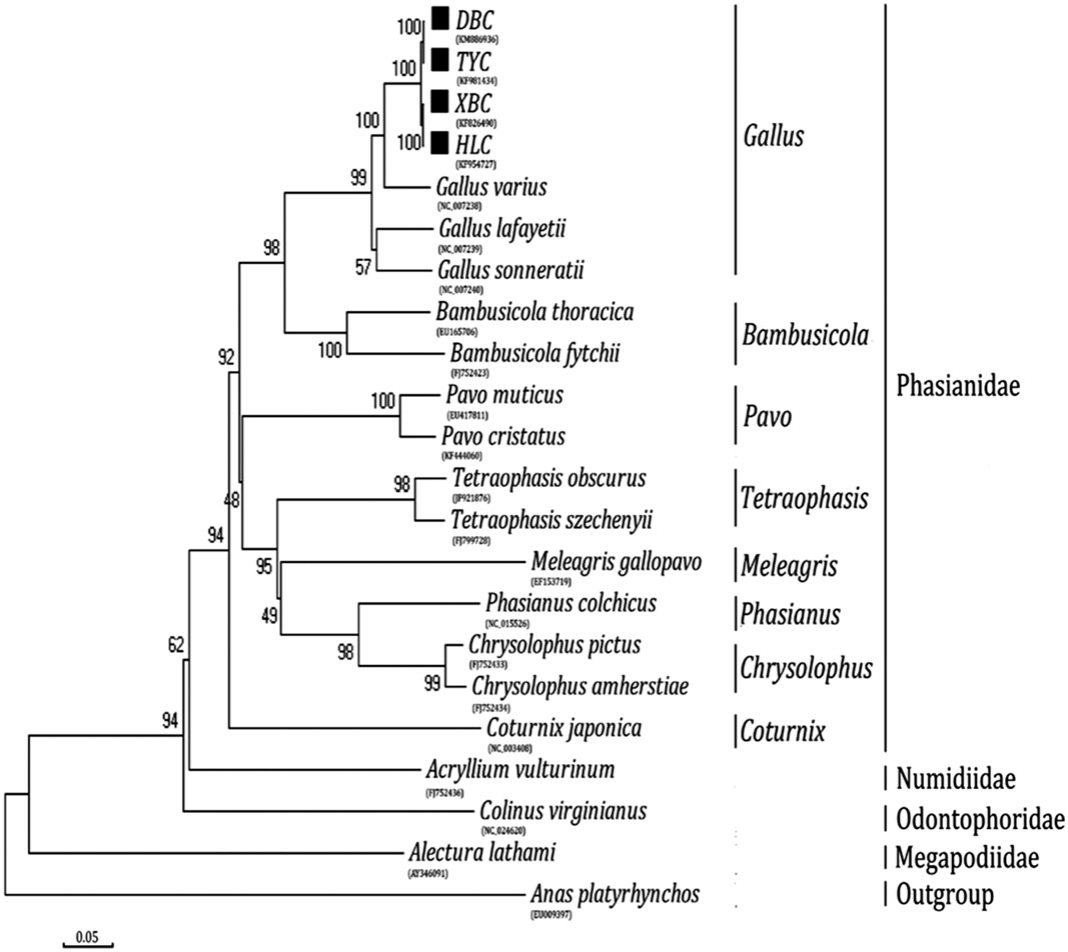
Phylogenetic analysis based on complete mitochondrial genome sequences. An N-J tree was built based on the phylogenetic analysis of 21 Galliformes species’ complete mitochondrial genomes. The mitochondrial genome sequences of the Galliformes species were obtained from the GenBank databases (Accession numbers have marked on the figure). Abbreviation of species indicates: DBC: Dongan black chicken; TYC: Taoyuan chicken; XBC: Xuefeng black-boned chicken; HLC: Huang Lang chicken.
